# Junctional bradycardia is a potential risk factor of stroke

**DOI:** 10.1186/s12883-016-0645-9

**Published:** 2016-07-25

**Authors:** Gwang Sil Kim, Jae-Sun Uhm, Tae-Hoon Kim, Hancheol Lee, Junbeom Park, Jin-Kyu Park, Boyoung Joung, Hui-Nam Pak, Moon-Hyoung Lee

**Affiliations:** Department of Cardiology, Severance Cardiovascular Hospital, Yonsei University College of Medicine, 50-1 Yonsei-ro Seodaemun-gu, Seoul, Republic of Korea

**Keywords:** Junctional bradycardia, Thromboembolic events, Stroke

## Abstract

**Background:**

This study aimed to determine the risk of thromboembolic events in patients with junctional bradycardia(JB).

**Methods:**

We retrospectively reviewed electrocardiograms(ECGs) for 380,682 patients. Those with JB on an ECG at least twice over a ≥3-month interval were included for analysis. We additionally included 138 CHADS_2_ score-matched patients(age, 68.4 ± 15.7 years; male, 52.2 %) in sinus rhythm as a control group. Between the JB patients(with or without retrograde P wave) and controls, we compared incidences of ischemic stroke and a composite of ischemic stroke, renal infarction, ischemic colitis, acute limb ischemia, and pulmonary embolism.

**Results:**

Among 380,682 patients (age, 47.6 ± 19.9 years; male, 49.3 %), 69 patients (age, 68.5 ± 16.5 years; male, 50.7 %) exhibited JB on an ECG at least twice over a ≥3-month interval; the overall prevalence of JB was 0.02 %. The mean follow-up period was 27.2 ± 26.2 months. Forty-five patients (65.2 %) in the JB group had no retrograde P wave. Ischemic stroke incidence was significantly higher in JB patients without a retrograde P wave than in controls (6/45 patients [13.3 %] and 3/138 patients [2.2 %], respectively; *P* = 0.007). The incidence of composite thromboembolic events was also significantly higher in JB patients without a retrograde P wave than in controls (8/45 patients [17.8 %] and 4/138 patients [2.9 %], respectively; *P* = 0.011). In a Cox proportional hazards model, JB patients without a P wave showed a greater incidence of stroke (hazard ratio, 8.89 [2.20–33.01], *P* = 0.007) than controls and JB patients with a P wave.

**Conclusions:**

Junctional bradycardia is potentially associated with ischemic stroke, particularly in the absence of an identifiable retrograde P wave.

## Background

Junctional bradycardia (JB) involves cardiac rhythms that arise from the atrioventricular junction at a heart rate of <60/min. In patients with retrograde atrioventricular nodal conduction, a retrograde P wave can be accompanied with JB. The event occurs as enhanced automaticity or as an escape rhythm during significant bradycardia with rates slower than the intrinsic junctional pacemaker [1]. JB can appear in patients with sick sinus syndrome or those with significant bradycardia that allows the atrioventricular nodal region to determine the heart rate [2]. As most of these patients are asymptomatic, there are no specific guidelines for evaluation and treatment. However, since there is no physiologic atrial contraction in JB, JB may be a potential cause of cardioembolic source [3, 4]. Here, we aimed to investigate associations between JB and thromboembolic events.

## Methods

We retrospectively reviewed electrocardiograms (ECGs) for 380,682 patients (47.6 ± 19.9 years; male, 49.3 %) from a single university hospital from January 2008 to December 2012. We screened consecutive patients who visited our outpatient center or were admitted to our hospital for ECG recording during the period. We initially excluded patients who were admitted due to acute myocardial infarction or acute stroke and patients whose life expectancy is less than 6 months due to cancer. A total of 972,683 ECGs (mean of 2.55 ECGs per patient) stored in our electronic database system (Muse System, General Electric Healthcare, Milwaukee, Wisconsin, USA) were included in this study. Twelve-lead ECGs were recorded by digital ECG instruments (General Electric Healthcare, Milwaukee, Wisconsin, USA). The digital sampling rate was 500 samples per second. The low and high cut-off frequencies were 0.5 and 100 Hz, respectively. Initially, the ECGs were screened by an automated ECG analysis algorithm embedded in our system to detect for JB. The algorithm for detecting JB is as follows: no P wave, a regular RR interval (i.e., an RR interval range that is less than 10 % of the average RR interval), narrow primary beat (<0.12 s for QRS duration), and a regular rate less than 60 beats/min. Then, three cardiologists reviewed the screened ECGs and finally selected those exhibiting JB in accordance with the following: JB was defined as a regular rhythm of less than 60 beats/min without a precedent P wave and a narrow QRS complex for a duration of less than 0.12 s. Retrograde P wave was defined as an inverted P wave after the QRS complex was noted. Respective ECGs with JB, with or without a retrograde P wave, were to be agreed upon by all three cardiologists. Inclusion criteria for analysis comprised age older than 18 years and JB on multiple ECGs at an interval of at least 3 months. Patients with transient JB and those with hyperkalemia at the time of ECG recording, history of end-stage renal disease, paroxysmal atrial fibrillation, and only one recording of JB were excluded. Additionally, patients who had been treated with implantation of a pacemaker or cardiac resynchronization therapy due to sick sinus syndrome or intractable heart failure were excluded from analysis.

Additionally, we also included 138 age-, gender-, and CHADS_2_ score-matched patients in sinus rhythm as a control group; these patients had undergone minor surgery, including nasal septal deviation and cataract surgery, in the same period and at the same hospital. For the controls, we excluded patients with a history of thromboembolic events, including ischemic stroke, and those lacking two-dimensional transthoracic echocardiographs. Since the incidence of JB was very low we aimed for 1:2 matching. In addition, to minimize confounding factors, we matched age, gender, and CHADS_2_ score, which are known as risk factors for stroke not only in patients with atrial fibrillation but also in patients without atrial fibrillation. CHADS_2_ score was calculated for each patient by assigning 1 point each for an age ≥75 years, history of heart failure, hypertension, and diabetes mellitus and 2 points for previous stroke or transient ischemic attack. Ischemic stroke was defined as symptomatic ischemic cerebral infarction with an apparent brain lesion on imaging studies. Beginning of follow-up was defined as time of acquisition of 2^nd^ ECG in JB group and acquisition of 1^st^ ECG in control group. Transient ischemic attack (TIA) was defined as a neurologist-confirmed transient episode of neurologic dysfunction without a brain lesion on imaging studies. Two-dimensional transthoracic echocardiography was performed using an ultrasound machine (Vivid 7, GE Medical Systems, Horten, Norway and Sonos 5500, Philips Medical Systems, Andover, Massachusetts, USA) with a 2.5-MHz transducer. Left atrial volume index was calculated by the prolate ellipsoid method [5, 6]. Left ventricular ejection fraction was measured using the modified Simpson’s method [5] on images of apical, 2- and 4-chamber views. Echocardiographic findings were reviewed by three cardiologists. Clinical characteristics; medication history; laboratory and imaging data; and development of ischemic stroke, transient ischemic attack, renal infarction, ischemic colitis, acute limb ischemia, and pulmonary embolism were reviewed from electronic medical records. JB was re-classified into two groups according to the presence of a retrograde P wave: JB without a retrograde P wave (JB without P) group and JB with a retrograde P wave (JB with P) group. The primary endpoint was ischemic stroke and transient ischemic attack. The secondary endpoint was a composite of ischemic stroke, transient ischemic attack, renal infarction, ischemic colitis, acute limb ischemia, and pulmonary embolism. We compared the incidences of the primary and secondary endpoints between the JB and control groups, as well as between the JB without P and JB with P groups. We calculated hazard ratios of JB for primary and secondary endpoints in comparison to sinus rhythm. We also calculated hazard ratios of JB without a retrograde P wave for primary and secondary endpoints in comparison to JB with a retrograde P wave. The study design was approved by the Institutional Review Board of Severance Hospital and conducted in compliance with the Declaration of Helsinki. The requirement for informed consent was waived, because of the retrospective nature of the study.

### Statistical analysis

Continuous variables are expressed as mean ± standard deviation, and categorical variables as numbers (percentages). Mann-Whitney and Fisher’s exact test were used for comparisons of continuous and categorical variables, respectively. Kaplan-Meier curves were constructed for time to event and compared by log-rank tests confined to 2 years of follow-up. Univariable and mutivariable Cox proportional hazards models were used to compare hazard ratios for primary and secondary endpoints between groups. The log-minus-log-survival function was used to assess the proportional hazards assumption and found that it was reasonable. Hazard ratios (HR) and 95 % confidence intervals (CI) were calculated. Two-sided *p* values <0.05 were considered statistically significant. The data were analyzed using the Statistical Package for the Social Sciences, version 20.0 (IBM Inc., Armonk, NY, USA).

## Results

### Population

The overall prevalence of JB for all 380,682 patients screened was 0.02 % in the present study. In total, 75 patients showed JB on ECGs twice over an interval of at least 3 months. Two patients had undergone implantation of pacemaker device and were excluded from analysis. An additional 4 patients who underwent cardiac surgery were excluded due to anticoagulation therapy that was maintained after valve surgery and clinical events that occurred during the immediate post-operative period. Finally, we included 69 patients (age, 68.5 ± 16.5 years; male, 50.7 %) with JB on ECGs. Among these, 40 patients (58 %) had been admitted; 29 patients (73 %) were admitted due to cardiovascular disease (14 patients with coronary artery disease, 7 patients with valvular heart disease, and 7 patients with myocardial disease). Of the 69 patients, three (4.3 %) had symptoms that might be associated with JB: two complained of orthostatic dizziness at an outpatient center. Their ECG revealed JB without a retrograde P wave. The other patient had a history of syncope, assumedly vasovagal syncope, and her ECG revealed JB with a retrograde P wave. These patients, however, did not receive any specific treatment, including pacemaker implantation. We also included 138 age-, gender-, and CHADS_2_ score-matched patients (age, 68.4 ± 15.7 years; male, 52.2 %) as a control group. Baseline characteristics for both groups are presented in Table 1. Thirty patients (43.5 %) underwent Holter monitoring and had no evidence of atrial fibrillation. The patients in the JB and control groups were followed for 27.2 ± 26.2 months and 25.4 ± 21.5 months, respectively, and 44.9 % of the patients had a CHADS_2_ score ≥2. Chronic kidney disease was significantly more frequent in the JB group than in the control group (*P* = 0.042). Left atrial volume index was significantly greater in the JB group than in the control group (*P* = 0.001). There were no significant differences in age, sex, comorbidities (hypertension, diabetes, heart failure, coronary artery disease, and stroke) or ejection fraction between the JB and control groups.

**Table 1:** Baseline characteristics

	JB group (*n* = 69)	Control group (*n* = 138)	*P* value*	JB with P group (*n* = 24)	JB without P group (*n* = 45)	*P* value^**^
Age (years)	68.5 ± 16.5	68.4 ± 15.7	0.593	67.3 ± 16.6	69.2 ± 16.5	0.273
Men, *n* (%)	35 (50.7)	72 (52.2)	0.883	10 (41.7)	25 (55.6)	0.318
Smoking, *n* (%)	26 (37.7)	40 (29.0)	0.210	8 (33.3)	18 (40.0)	0.614
Hypertension, *n* (%)	39 (56.5)	76 (55.1)	0.883	11 (45.8)	28 (62.2)	0.213
Diabetes mellitus, *n* (%)	30 (43.5)	50 (36.2)	0.364	8 (33.3)	22 (48.9)	0.308
Heart failure, *n* (%)	6 (8.7)	11 (8.0)	>0.999	3 (12.5)	3 (6.7)	0.412
Chronic kidney disease, *n* (%)	20 (29.0)	22 (15.9)	0.043	5 (20.8)	15 (33.3)	0.404
History of CAD, *n* (%)	21 (30.4)	44 (31.9)	0.875	3 (12.5)	18 (40.0)	0.027
History of stroke, *n* (%)	1 (1.4)	1 (0.7)	>0.999	0 (0.0)	1 (2.2)	>0.999
Ejection fraction (%)	64.2 ± 11.3	63.3 ± 11.0	0.485	63.8 ± 13.4	64.4 ± 10.1	0.772
LAVI (ml/m^2^)	45.1 ± 30.3	28.0 ± 11.3	0.001	40.8 ± 10.4	47.1 ± 36.1	0.740
Antiplatelet agents, *n* (%)	33 (47.8)	101 (73.2)	0.883	9 (37.5)	24 (53.3)	0.312
Statin, *n* (%)	26 (37.7)	52 (37.7)	>0.999	7 (29.2)	19 (42.2)	0.312
Anticoagulants, *n* (%)	8 (11.6)	6 (4.3)	0.091	2 (8.3)	6 (13.3)	0.704
CHADS_2_ score	1.48 ± 1.16	1.49 ± 1.19	0.991	1.17 ± 1.13	1.64 ± 1.15	0.103
0, *n* (%)	16 (23.2)	32 (23.2)		8 (33.3)	8 (17.8)	
1, *n* (%)	22 (31.9)	44 (31.9)		8 (33.3)	14 (31.1)	
2, *n* (%)	16 (23.2)	32 (23.2)		5 (20.8)	11 (24.4)	
3, *n* (%)	12 (17.4)	24 (17.4)		2 (8.3)	10 (22.2)	
4, *n* (%)	3 (4.3)	6 (4.3)		1 (4.2)	2 (4.4))	
Follow-up duration (month)	27.2 ± 26.2	25.4 ± 21.5	0.948	39.3 ± 36.5	20.7 ± 15.6	0.008

*CAD* coronary artery disease, *JB* junctional bradycardia, *LAVI* left atrial volume index

^*^comparison between the JB group and control group

^**^comparison between the JB without P group and JB with P group

### Thromboembolic events

Incidences of thromboembolic events are summarized in Table 2. Thromboembolic events occurred in 8 patients (17.8 %) in the JB without P group. In contrast, thromboembolic events occurred in one (4.2 %) and 4 (2.9 %) patients in the JB with P group and the control group, respectively. Stroke and composite thromboembolic events were significantly more frequent in the JB without P group than the control and JB with P groups. Six patients (13.3 %) developed stroke in the JB without P group, and the time interval between detection of JB and the date the events occurred ranged from 1 month to 4 years. Among 6 patients, three had undergone cardiac surgery (coronary artery bypass graft in 2 patients and mitral valve repair in 1 patient). Their clinical events occurred 40 months, 31 months, and 7 months after cardiac surgery. We summarized the clinical history of patients who showed thromboembolic events in Table 3. We could not compare the incidences of renal infarction, ischemic colitis, acute limb ischemia, and pulmonary embolism due to their rare incidence. Using the Cox proportional hazards model, JB without retrograde P wave was significantly associated with both stroke and composite thromboembolic events, even after adjusting for confounding clinical factors, such as gender and CHADS_2_ score, as described on Table 4. After excluding TIA, JB without retrograde P wave was also associated with incidence of stroke in multivariable analysis, which included gender and CHADS_2_ score (hazard ratio, 5.062 [2.12–22.63], *P* = 0.034).

**Table 2:** Thromboembolic events

	JB with P group (*n* = 24)	JB without P group (*n* = 45)	Control group (*n* = 138)	*P* value	*P* value
Stroke (%)	0	4 (8.9)	3 (2.2)		–
TIA (%)	0	2 (4.4)	0		
Stroke and TIA (%)	0	6 (13.3)	3 (2.2)	0.007^*^	
Renal infarction, *n* (%)	1 (4.2)	0	0	–	–
Ischemic colitis, *n* (%)	0	0	1 (0.7)	–	–
Acute limb ischemia, *n* (%)	0	1 (2.2)	0	–	–
Pulmonary embolism, *n* (%)	0	1 (2.2)	0	–	–
Composite thromboembolic events, *n* (%)	1 (4.2)	8 (17.8)	4 (2.9)	0.011^**^	0.059^***^

*JB* junctional bradycardia*TIA* transient ischemic attack

^*^comparison between the JB without P group and control group

^**^comparison among the JB without P group, JB with P group, and control group

^***^comparison between the JB without P group and JB with P group

**Table 3:** Clinical information in patients with thromboembolic events

Patients number	Age	Sex	Group	Heart rate (/min)	Thromboembolic events	Underlying disease
1	74	Female	Control	70	Stroke	HTN, CAD
2	74	Female	Control	66	Stroke	HTN, DM
3	80	Male	Control	80	Ischemic colitis	HTN, DM
4	79	Male	Control	56	Stroke	HTN, DM, CAD
5	65	Female	JB with P	40	Renal infarction	HTN
6	66	Female	JB without P	40	TIA	none
7	73	Male	JB without P	46	PAD	HTN, CAD (s/p CABG)
8	80	Male	JB without P	50	Pulmonary embolism	HTN, DM, CKD, CAD (s/p CABG)
9	70	Male	JB without P	48	Stroke	HTN, DM, s/p MVR
10	86	Male	JB without P	50	TIA	HTN
11	80	Male	JB without P	52	Stroke	HTN, DM, CKD (s/p kidney transplantation), CAD
12	78	Female	JB without P	56	Stroke	HTN, DM, CAD
13	88	Female	JB without P	46	Stroke	HTN, DM, CKD, CAD

*CABG* coronary artery bypass graft surgery, *CAD* coronary artery disease, *CKD* chronic kidney disease, *DM* diabetes mellitus, *HTN* hypertension, *JB* junctional bradycardia, *LAD* left anterior descending, *MVR* mitral valve repair, *P* P wave, *PAD* peripheral artery disease, *SCD* sudden cardiac death, *TIA* transient ischemic attack

**Table 4:** Independent predictors of thromboembolic events

	Univariable		Mutivariable	
	HR (95 % CI)	*P* value	HR (95 % CI)	*P* value
a. Stroke and transient ischemic attack				
Male	0.58 (0.14–2.34)	0.472		
Chronic kidney disease	1.95 (0.48–7.82)	0.352		
CHADS_2_ score ≥2	5.74 (1.17–28.25)	0.032	4.67 (0.96–22.73)	0.062^***^
LAVI (ml/m^2^)	1.01 (0.97–1.06)	0.562		
JB^*^	2.60 (0.59–11.53)	0.210		
JB without P^**^	6.56 (1.61–26.75)	0.007	8.89 (2.20–33.01)	0.002^***^
b. Composite thromboembolic events				
Male	0.99 (0.36–3.27)	0.993		
Chronic kidney disease	1.75 (0.54–5.70)	0.352		
CHADS_2_ score ≥2	3.44 (1.04–11.33)	0.043	4.07 (1.11–14.87)	0.031^***^
LAVI (ml/m^2^)	1.01 (0.99–1.03)	0.391		
JB without P^**^	5.69 (1.84–17.63)	0.003	5.04 (1.68–15.09)	0.004^***^

*JB* junctional bradycardia, *LAVI* left atrial volume index, *P* P wave

^*^comparison with the control group

^**^comparison with the control group and JB with P group

^***^adjustment for male sex, CHADS_2_ score ≥2, and JB without P group

**Fig. 1:**
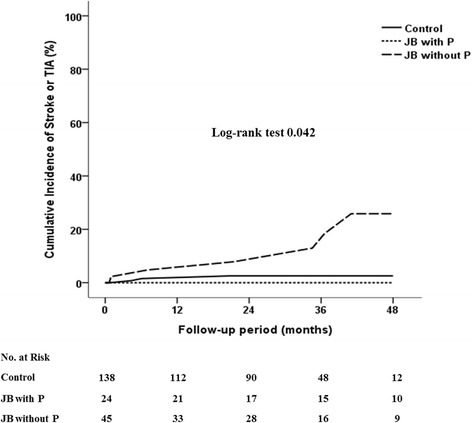
Kaplan-Meier curve of the incidence of stroke at 48 months follow-up after the detection of junctional bradycardia. JB = junctional bradycardia; P = P wave; TIA = transient ischemic attack

We compared the incidence of stroke or transient ischemic attack among the three groups using Kaplan-Meier curves over 48 months of follow-up, as shown in Fig. 1. A difference in event-free survival was noted in the early period of follow-up and did not decrease after follow-up for up to 48 months.

## Discussion

In the present study, JB was found to be potentially associated with the occurrence of a composite of thromboembolic events. JB without a retrograde P wave was also shown to be a significant risk factor for both the occurrence of stroke and the composite of thromboembolic events.

Paroxysmal atrial fibrillation and sick sinus syndrome have been reported as risk factors for thromboembolic events, such as acute stroke [4, 7–9]. Paroxysmal atrial fibrillation is considered an earlier form of arrhythmia, showing less electrical and structural remodeling of the atria; nonetheless, the risk of thromboembolic events is similar for persistent or permanent atrial fibrillation. Accordingly, most current guidelines recommend the use of oral anticoagulation for patients with stroke risk factors, irrespective of the type of atrial fibrillation [10, 11]. Sinus node dysfunction is also reported as a risk factor for stroke [3, 12]. The annual incidence of stroke in patients with sick sinus syndrome is estimated at approximately 6–10 %, even after pacemaker implantation and physiologic pacing, such as DDD mode [4, 9, 13]. Virchow described a triad of abnormalities associated with thrombus formation: abnormal vessel wall, abnormal blood constituents, and abnormal flow [14]. Of these, ineffective atrial contraction could be associated with abnormal flow. Atrial performance is categorized into four phases: reservoir function, conduit function, active contractile pump function, and suction force [15]. In late diastole, the atrium behaves as a pump as pressure rises due to active atrial contraction and pushes the blood through the mitral valve, contributing 15–30 % to left ventricular filling [16, 17]. In a case report of thromboembolism in a patient with intermittent periods of sinus arrest and junctional escape bradycardia, spontaneous echo contrast was detected on transthoracic echocardiography [13]. Additionally, Vincelj et al. reported that spontaneous echo contrast is associated with left atrial enlargement, which is known as a risk factor for thromboembolism [18]. Thus, spontaneous echo contrast suggested that inactive atrial pumping might cause blood stagnation and could be associated with cardioembolic embolism.

We hypothesized that JB would show similar pathophysiology to atrial fibrillation and sick sinus syndrome in terms of ineffective atrial contraction, which may be a cardiogenic thromboembolic source. Our data indicated that JB without retrograde P wave is a robust risk factor for both stroke and thromboembolic events in multivariable analysis. However, the main pathogenic mechanism for thromboembolic events is not well understood. In 3 patients who experienced thromboembolic events in the JB without P group, thromboembolic events did not occur in the immediate postoperative period. Therefore, the events could not be complications associated with cardiac surgery.

Additionally, in the present study, left atrial volume index (LAVI) was significantly larger in the JB group (45 ml/m^2^ vs. 28 ml/m^2^) than in the control group. The left atrium is highly susceptible to acute and chronic stress factors, such as alterations in both preload and afterload [19]. Reportedly, left atrial volume index is a more accurate parameter for left atrial size than left atrial diameter. Previously, Corbalan et al. reported that left atrial enlargement on two-dimensional echocardiography is a significant independent risk factor for systemic embolism in patients with symptomatic paroxysmal atrial fibrillation [20]. In addition to patients with atrial fibrillation, LAVI has also been shown to be associated with stroke and cardiac thrombi in patients with sinus rhythm or without atrial fibrillation [21, 22]. Thus, in the JB group, large left atrial volume index could be associated with loss of atrial kick, which might contribute to increased preload of the left atrium.

We used the CHADS_2_ scoring system to control for confounding factors in predicting the risk of stroke based on junctional rhythm, since the CHADS_2_ scheme has been reported as a reliable predictive parameter in patients without atrial fibrillation [23, 24]. CHADS_2_ score did not predict stroke or thromboembolic events even in subgroup analysis (JB without retrograde P wave), unlike previous reports in which CHADS_2_ and CHA_2_DS_2_-VASc scores were associated with an increased risk of stroke in patients with paced sick sinus syndrome [7]. This difference between results could be explained by variation in the study populations.

When patients with JB have co-morbidities that are known risk factors for embolic events, preventive strategies, such as anticoagulant administration or pacemaker implantation, might be considered. However, a randomized controlled study with a large population is also needed to confirm these findings.

### Limitations

There are some limitations to our study. First, it is a retrospective study of a relatively small sample size conducted in a single center. Furthermore, continuous ECG monitoring, such as Holter monitoring, was only performed in about 43 % of the patients with JB; therefore, we could not guarantee the absence of asymptomatic paroxysmal atrial fibrillation in patients without Holter monitoring. Furthermore, because of the very low prevalence of JB and very small number of clinical events, it would be difficult to conclude a robust correlation between JB and clinical events such as stroke. Thus, a large-scale, prospective, observational study is being contemplated.

## Conclusions

Junctional bradycardia is potentially associated with ischemic stroke, particularly in the absence of an identifiable retrograde P wave.

## Abbreviations

CI, confidence interval; ECG, electrocardiogram; HR, heart rate; JB with P, junctional bradycardia with P wave; JB without P, junctional bradycardia without P wave; JB, junctional bradycardia; LAVI, left atrial volume index; TIA, transient ischemic attack
